# Effects of *Mactra chinenesis* Peptides on Alcohol-Induced Acute Liver Injury and Intestinal Flora in Mice

**DOI:** 10.3390/foods13101431

**Published:** 2024-05-07

**Authors:** Dong Wu, Ming Cheng, Xiangzhou Yi, Guanghua Xia, Zhongyuan Liu, Haohao Shi, Xuanri Shen

**Affiliations:** 1 School of Food Science and Engineering, Hainan University, Haikou 570228, China; wudong1997@hainanu.edu.cn (D.W.); chengmingmvp@163.com (M.C.); xiangzhouyi1995@hainanu.edu.cn (X.Y.); xiahuanghua@hainanu.edu.cn (G.X.); liuzy@hainanu.edu.cn (Z.L.); 2 Key Laboratory of Food Nutrition and Functional Food of Hainan Province, Haikou 570228, China; 3 Hainan Engineering Research Center of Aquatic Resources Efficient Utilization in South China Sea, Hainan University, Haikou 570228, China; 4 School of Food Science and Engineering, Hainan Tropical Ocean University, Sanya 572022, China

**Keywords:** *Mactra chinenesis*, bioactive peptides, lipid metabolism, inflammatory responses, hepatoprotective effect

## Abstract

Food-borne bioactive peptides have shown promise in preventing and mitigating alcohol-induced liver injury. This study was the first to assess the novel properties of *Mactra chinenesis* peptides (MCPs) in mitigating acute alcoholic liver injury in mice, and further elucidated the underlying mechanisms associated with this effect. The results showed that MCPs can improve lipid metabolism by modulating the AMPK signaling pathway, decreasing fatty acid synthase activity, and increasing carnitine palmitoyltransferase 1a activity. Meanwhile, MCPs ameliorate inflammation by inhibiting the NF-κB activation, leading to reduced levels of pro-inflammatory cytokines (tumor necrosis factor-α and interleukin-1β). Additionally, a 16S rDNA sequencing analysis revealed that MCPs can restore the balance of gut microbiota and increase the relative abundance of beneficial bacteria. These findings suggest that supplementation of MCPs could attenuate alcohol intake-induced acute liver injury, and, thus, may be utilized as a functional dietary supplement for the successful treatment and prevention of acute liver injury.

## 1. Introduction

Excessive alcohol consumption can be extremely damaging to a person’s physical and mental health. Recent studies have shown that 3.3 million people die globally each year from excessive alcohol consumption, accounting for 6% of all deaths worldwide [1]. The liver is the principal organ responsible for 60 to 90% of the metabolism of alcohol and is the organ that alcohol intoxication mostly affects [2]. Acute alcohol-induced liver injury is liver damage resulting from excessive alcohol consumption over a short duration [3,4]. Initially, it mainly manifests as lipid accumulation and degeneration in the liver, and then gradually develops into steatohepatitis, cirrhosis, and even hepatocellular carcinoma [5,6]. With the aggravation of liver damage, the above forms can exist separately or simultaneously [7]. As such, liver protection is an increasingly emphasized aspect of healthcare in contemporary times.

The main risk factors associated with the development of alcohol-induced damage include excessive blood ethanol concentrations and its metabolic intermediates (acetaldehyde, reactive oxygen species, etc.) [8]. Acetaldehyde produced by the metabolism of ethanol in hepatocytes by ethanol dehydrogenase not only directly affects protein function and DNA damage repair, but also induces oxidative stress, lipid accumulation, and inflammatory responses that cause different degrees of liver injury [9]. Many data have emerged that indicate that the symptoms of ALD could be alleviated through regulating some signaling pathways, such as the NF-κB, mitogen-activated protein kinase (MAPK), and AMP-activated protein kinase (AMPK) signaling pathways. Currently, effective ways to treat alcohol-induced liver injury include abstinence, drug therapy, nutritional therapy, and combination therapy [10]. Among them, abstinence from alcohol is the key to treatment, and while in early prevention and treatment nutritional support can improve liver function to a certain extent, drugs must be used to alleviate symptoms in severe cases. However, due to the potentially toxic side effects of drugs and the high dosage required to maximize the effect of drugs, the liver will be overloaded [11]. In order to address the current limitations in effectively treating alcohol-induced liver injury, natural hepatoprotective agents have attracted widespread attention.

Numerous studies have shown that bioactive substances such as flavonoids, polyphenols, polysaccharides, and bioactive peptides can reduce alcoholic liver injury [12,13,14,15]. Bioactive peptides produced from wheat, maize, and mushrooms have been identified as potentially effective hepatoprotective agents throughout the last ten years [16,17,18]. Since marine species account for nearly half of the total global biodiversity, in recent years researchers have begun to emphasize the use of various marine protein resources to prepare bioactive peptides for food and pharmaceutical applications. Marine-derived bioactive peptides offer numerous advantages, including high protein content, exceptional nutritional value, potent biological activity, non-antigenicity, minimal toxic side effects, ease of modification, and long-term suitability for consumption. According to reports, marine bioactive peptides extracted from oysters [19], tilapia fish skin [20], and crucian carp swim bladder [21] have shown potential for treating alcohol-induced liver injury.

*Mactra chinenesis* (MC) is a shellfish that predominantly inhabits the shallow waters of the Yellow Sea. Originating from Dandong in China, it is recognized for its distinctive yellow shells and flesh. The muscle of MC is rich in protein, multivitamins, and nutrients needed by the human body. Studies have shown that the compounds found in MC, such as proteins and glycine, may have protective effects on the liver [22,23]. Furthermore, because MC is a strong source of bioactive peptides, it shows promise in preventing alcohol-induced liver damage. At present, little is known about the effect and mechanism of MC on alcohol-induced liver injury. In order to prevent alcoholic liver damage, the aim of this study was to evaluate MC’s potential as a valuable source of bioactive peptides. MC was subjected to hydrolysis by alkaline protease to produce *Mactra chinenesis* peptides (MCPs), and its molecular weight and amino acid content were determined using high-performance liquid chromatography (HPLC). In order to explore the mechanism of MCPs for sobriety and liver protection, real-time fluorescence quantitative polymerase chain reaction (qRT-PCR) and Western blotting were used to examine the expression levels of genes and proteins involved in alcohol metabolism, lipid metabolism, and inflammation. This work provides a solid theoretical basis for the practical use of MCPs in the production of health-food items, as well as for their use in reducing alcohol-induced liver damage.

## 2. Materials and Methods

### 2.1. Materials

*Mactra chinenesis* was purchased from a seafood supermarket (Lianyungang, China). Commercial kits for the determination of alcohol dehydrogenase (ADH), aldehyde dehydrogenase (ALDH), total cholesterol (TC), triacylglyceride (TG), alanine aminotransferase (ALT), serum aspartate aminotransferase (AST), superoxide dismutase (SOD), and malondialdehyde (MDA) were purchased from Grace Biotechnology Co., Ltd. (Suzhou, China). Detection kits for quantification of interleukin-1β (IL-1β) and tumor necrosis factor-α (TNF-α) were obtained from Xinyu Biotechnology Co., Ltd. (Shanghai, China). Antibodies against phospho-adenosine 5′-monophosphate (AMP)-activated protein kinase (p-AMPK), toll-like receptor 4 (TLR4), cytochrome P450 2E1 (CYP2E1), myeloid differential protein-88 (MyD88), peroxisome proliferator-activated receptor-α (PPAR-α), carnitine palmitoyltransferase 1a (Cpt1a), sterol regulatory element binding protein 1c (SREBP-1c), and interleukin-6 (IL-6) were bought from Servicebio Technology Co., Ltd. (Wuhan, China). A protein content determination kit was offered by Beyotime Biotechnology Co., Ltd. (Shanghai, China). Chinese spirits (alcohol content of 56%) were provided by Red Star Co., Ltd. (Beijing, China). Other chemical reagents were of analytical grade.

### 2.2. Preparation of MCP

The frozen MC was allowed to thaw overnight at 4 °C and was homogenized at 10,000 g (IKA-Werke GmbH & Co. KG, Staufen, Germany). Defatting was performed by adding anhydrous ethanol at a ratio of 1:4 (*w/v*), followed by sonication at 250 W, 40 kHz, and 55 °C for 1.5 h (SB-5200D, Scientz Biotechnology Co., Ltd., Ningbo, China). After discarding the filtrate, the leftover solution was blended at a 1:3 (*w/v*) ratio with deionized water. Alkaline protease (5 kU/g) was added and incubated on a constant temperature shaker at 55 °C and 125 g for 12 h. By continually injecting 1M NaOH, the pH was maintained at 9.5 throughout the procedure. To inactivate the enzyme, the combinations were then cooked for 15 min in boiling water. Following the mixture’s cooling to ambient temperature, it was centrifuged for 20 min at 4 °C at 8000× *g* (ST 16R, Thermo Scientific, MA, USA). The supernatant was evaporated using a rotary evaporator (Yarong RE-5298, Shanghai, China), concentrated, and freeze-dried to obtain the MCP, which was then stored at −20 °C for later use.

### 2.3. Determination of Molecular Weight Distribution and Total Amino Acid Composition

A total of 100 mg of sample was placed in a 10 mL volumetric flask, diluted to volume with mobile phase, and then filtered through a 0.45 μm microporous filter membrane prior to injection. A Waters 1525 HPLC system (Waters, MA, USA) equipped with a TSKgel 2000 SWXL column (300 mm × 7.8 mm, Tosoh, Japan) was applied to determine the molecular weight distribution of the MCPs. At a flow rate of 0.5 mL/min, peptides were eluted using a mobile phase consisting of acetonitrile, water, and trifluoroacetic acid (45:55:0.1, *v/v/v*), and they were detected at 220 nm. Four standards, including cytochrome C (12400 Da), mycobacteriophage (1450 Da), ethionine-ethionine-tyrosine-arginine (451 Da), and ethionine-ethionine-ethionine (189 Da) were used to calculate the molecular weight distribution.

The total amino acid content was determined using an Agilent 1260 HPLC system equipped with a Hypersil AA-ODS column (200 mm × 2.1 mm, 5 μm). The reaction column temperature was set at 115 °C, the separation column temperature at 70 °C, the buffer flow rate at 240 uL/min, and the detection wavelengths at 570 nm and 440 nm. Briefly, an appropriate amount of the samples was weighed and mixed with 5 mL of 6M HCl. The mixture was sealed and hydrolyzed at 110 °C for 24 h, followed by dilution to 25 mL with double-distilled water. Subsequently, 1 mL of the liquid was dried at 60 °C and then reconstituted to the desired concentration with diluent for analysis. For quantification, a total of 17 amino acids were employed as external standards.

### 2.4. Measurement of MCP-Mediated Liver-Protective Effects in Mice

#### Experimental Animals and Treatment

Healthy male specific pathogen-free (SPF) KM mice (8 weeks old, body weight 35 g ± 3 g) were provided by Hunan Slrc Jingda Laboratory Animal Co., Ltd. Hainan University Animal Ethics Committee granted approval for the animal study (Approval No.: HNUAUCC-2021-00046). All procedures were carried out in accordance with the “Guidelines for the Management and Use of Laboratory Animal Husbandry at Hainan University”. The animals were kept in a controlled environment with a 12 h light/dark cycle, a constant temperature of 25 ± 2 °C, relative humidity of 60% ± 10%, and free access to food and water.

After receiving acclimation food for a week, mice (*n* = 10) were allocated at random to one of seven groups: normal control (NC), alcohol-induced model (AM), positive control (PC, 600 mg/kg·bw), oyster peptide (OP, 600 mg/kg·bw), the low-dose MCP (MCP-L, 100 mg/kg·bw), the median-dose MCP (MCP-M, 600 mg/kg·bw), and the high-dose MCP (MCP-H, 900 mg/kg·bw) groups. Herein, King Drink, used as a PC group, is a commercialized dietary supplement containing oyster extract, soy peptide powder, L-carnitine, vitamin C, taurine, and L-cysteine to promote alcohol metabolism in the human body. NC and AM groups were administered with the same volume of saline solution. In addition to the NC group receiving normal saline, all other groups were gavaged with white wine (10 mL/kg·bw, 56% alcohol) every 24 h for a total of three times. As a preventive measure, King Drink, MCP, and OP were gavaged separately 30 min before each alcohol intake.

Prior to each gavage, the mice’s body weights were assessed in order to calculate the precise amount of alcohol or sample that would be consumed. Following their initial alcohol treatment, the mice’s intoxicated behavior was evaluated using tests for the loss of righting reflex (LORR). The mice’s blood was taken 12 h after the last treatment was finished. The livers were removed from the mice and preserved at −80 °C for use in future research when the animals were killed via cervical dislocation.

### 2.5. LORR and Liver Index Assay

The LORR test was used to assess the mice’s tolerance to acute alcohol-induced intoxication [24]. In this study, the incapacity of mice to self-correct within 30 s of inversion was designated as LORR. The latency of LORR was determined as the amount of time that passed after alcohol was administered before LORR started to manifest. The duration of LORR was from the onset of LORR to recovery.

The following formula was used to determine the mice liver index:

(1) $$Mice\;liver\;index\;(g/kg)=\frac{Liver\;weight}{Body\;weight}$$

### 2.6. Determination of Biochemical Indicators

Serum was extracted from the supernatant after the drawn blood was centrifuged for ten minutes at 4 °C at 3000× *g*. The levels of ALT, AST, TC, TG, TNF-α, and IL-1β in serum were measured in accordance with the commercial kit’s instructions.

Liver tissue and pre-cooled saline were mixed at a ratio of 1:9 (m:v) and homogenized at 4 °C. The supernatant was then extracted by centrifugation at 12,000× *g* for 15 min at 4 °C. The levels of ADH, ALDH, MDA, SOD, and TG in the liver were measured by a detection kit.

### 2.7. Histopathologic Analysis

The freshly harvested liver tissues were fixed in 4% paraformaldehyde, dehydrated, and embedded in paraffin. Then, 5 μm sections were cut and stained with hematoxylin and eosin (H&E) for histopathological analysis. In addition, Oil Red O dye was used to stain the frozen liver slices, and the samples were inspected under a light microscope (Nikon Eclipse Ci-L, Tokyo, Japan).

### 2.8. qRT-PCR Assay

The qRT-PCR technique was utilized to determine the mRNA amplification levels of AMPK, SREBP-1c, FAS, CYP2E1, PPAR-α, TNF-α, MyD88, NF-κB p65, Cpt1a, TLR4, and IL-6 in the liver. In brief, an Eastep Super Total RNA Extraction kit was used to extract all of the RNA from the tissues in accordance with the manufacturer’s instructions (Promega Biotech Co., Ltd., Beijing, China). Next, the RevertAid First Strand cDNA Synthesis Kit (Thermo Fisher Scientific, MA, USA) was used to reverse-transcribe into cDNA. For the qRT-PCR, the CFX Connect Real-Time PCR Detection System (Bio-Rad, Hercules, CA, USA) was utilized. The relative expression of the target gene mRNA was measured using the 2^−ΔΔCt^ technique, with GAPDH acting as the internal reference. The primer sequences that were used in this study are described in Table S2 and were obtained from Sangon Biotech (Shanghai, China).

### 2.9. Western Blotting

The expression levels of AMPK, SREBP-1c, PPAR-α, Cpt1a, TLR4, MyD88, NF-κB p65, TNF-α, and IL-6 in the liver were tested based on Western blotting. Proteins were extracted from livers using a lysis buffer (Servicebio, Wuhan, China) and centrifuged (5000× *g*, 4 °C, 20 min). Using a BCA assay kit (Beyotime, Shanghai, China), the amount of protein in the supernatant was determined. SDS-polyacrylamide gel electrophoresis (EpiZyme, Shanghai, China) was used to separate equal quantities of protein, which were then transferred into PVDF membranes, blocked with 5% skim milk, and incubated with certain primary antibodies for 12 h. A secondary antibody that had been labeled with peroxidase was then incubated with the membranes. The Tanon-5200 multipurpose imaging system (Shanghai, China) was used to observe the blots after they were identified using an improved chemiluminescence detection kit (Biosharp, Anhui, China).

### 2.10. High-Throughput Sequencing of Intestinal Flora

To extract DNA from the colonic contents, the QIAamp DNA Stool Mini Kit (QIAGEN, Duesseldorf, Germany) was utilized. The quality of the DNA was assessed for concentration and integrity through analysis using 1% agarose gel electrophoresis. The structure of the intestinal microbiota was analyzed based on the V3–V4 variable region of the bacterial 16S rRNA gene. Following quantification and purification of PCR products, sequencing was performed on the Illumina HiSeq platform.

### 2.11. Statistical Analysis

Results were expressed as mean ± standard error of the mean (SEM) and analyzed by one-way analysis of variance (ANOVA) or t-test using SPSS software (version 26.0, SPSS, Inc., Chicago, IL, USA). For differences that were considered significant or highly significant, the crucial *p*-values were set at 0.05 and 0.01.

## 3. Results

### 3.1. Molecular Weight Distribution and Amino Acid Compositions of Extracted Peptides

As shown in Table 1, the average molecular weights of MCPs and OPs were 498 Da and 435 Da, respectively. The molecular weights of both peptides were primarily less than 1 kDa, with MCPs accounting for 94.25% and OP for 92.24%. The content of small molecule peptides (molecular weight < 1 kDa) in the MCPs was higher than that in the OP.

As presented in Table 2, the total amino acid analysis revealed that the MCPs were rich in amino acids, and their essential amino acid content reached 42.98%, which was slightly higher than that of the OP. The proportion of branched-chain amino acids in the MCPs was 19.90%. Additionally, the MCPs contained a high amount of hydrophobic amino acids, including Gly (8.86 ± 0.55 g/100 g), Ala (9.36 ± 0.69 g/100 g), Val (3.18 ± 0.12 g/100 g), Met (6.19 ± 0.49 g/100 g), Phe (5.35 ± 0.38 g/100 g), Ile (3.01 ± 0.12 g/100 g), Leu (13.71 ± 0.83 g/100 g), and Pro (0.84 ± 0.13 g/100 g).

### 3.2. Effects of MCPs on Acute Alcohol Intoxication Tolerance and Body Weight in Mice

Alcohol may impact the central nervous system and result in various behavioral problems, including LORR. As shown in Table 3, LORR occurred in the model group of mice within approximately 29.39 min after ingesting alcohol and lasted 434.83 min. The PC, OP, and MCP groups significantly increased the latency of LORR and decreased the duration of LORR in the mice (*p* < 0.05), and there was a dose effect among the MCP groups. Among them, LORR occurred in the MCP-H group at about 42.21 min after alcohol intake, and the LORR duration was 312.40 min.

Body weight change in mice is one of the important indicators of subacute toxicity. It can reflect the development status of various organ systems and the increase or decrease in food intake. At the end of the experiment, compared with the NC group, the body weight of mice in the AM group was significantly reduced. The body weight of mice in the MCP-H group increased significantly compared to the AM group (*p* < 0.05). In addition, excessive alcohol consumption will increase the alcohol content in the body and lead to liver enlargement. The degree of liver damage can be indicated by the liver index, one of the markers of alcoholic liver impairment. Mice in the AM group had considerably greater liver weights and liver indices than mice in the NC group (*p* < 0.05). “King Drink”, a commercially available liver protectant, alleviated this symptom. Meanwhile, similar liver protection results occurred in the OP and MCP groups. By pre-administering the supplement, the MCP-H group significantly alleviated the alcohol-induced liver swelling and increased liver index.

### 3.3. Effects of MCPs on the ALT and AST Levels in Serum

**Figure 1:**
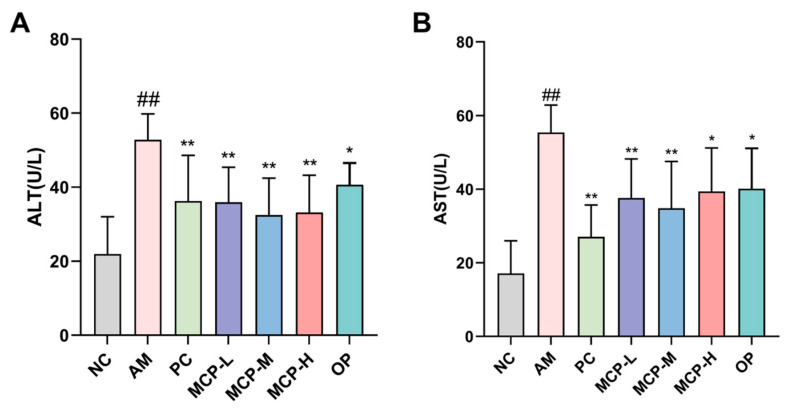
Effects of MCPs on the activity of ALT (**A**) and AST (**B**) in mice. Data are expressed as the means ± SEM (n = 10). Compared with the NC group: ## *p* < 0.01; compared with the AM group: * *p* < 0.05 and ** *p* < 0.01.

The effects of MCPs on ALT and AST levels are illustrated in Figure 1. In comparison to the NC group, the ALT levels in the AM group were significantly elevated (*p* < 0.01), rising from 21.96 to 52.77 U/L, while the AST levels increased from 17.11 to 55.42 U/L. Compared with the AM group, the serum AST and ALT levels in the OP and MCP groups of mice significantly decreased, especially the ALT levels in all dosage groups of MCPs (*p* < 0.01). Similarly, the serum AST levels in the MCP-L and MCP-M groups were markedly reduced (*p* < 0.01). The above results implied that MCPs improved liver function in acute alcohol-induced liver injury mice to a certain extent. Its mitigating effect was superior to that of the OP, the raw material of most hangover products on the market.

### 3.4. Effects of MCPs on Alcohol-Metabolizing Enzymes

**Figure 2:**
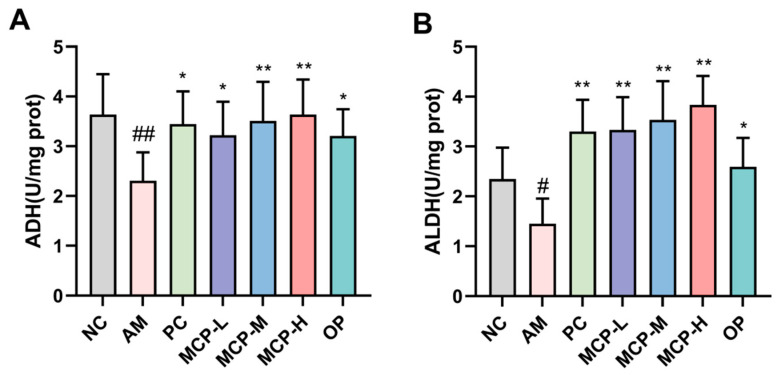
Effects of MCPs on liver alcohol-metabolizing enzymes activities of ADH (**A**) and ALDH (**B**) in mice. Data are expressed as the means ± SEM (n = 10). Compared with the NC group: # *p* < 0.05 and ## *p* < 0.01; compared with the AM group: * *p* < 0.05, ** *p* < 0.01.

The majority of alcohol that enters the bloodstream is metabolized in the liver through the ADH pathway, which entails the participation of both ADH and ALDH enzymes. The effects of MCPs on the activities of ADH and ALDH in the mouse liver are shown in Figure 2. In comparison to the NC group, the activities of ADH and ALDH in the livers of mice in the AM group decreased by 57.73% and 61.78%, respectively. However, compared to the AM group, MCP pretreatment exhibited a dose-dependent reversal of these outcomes. More specifically, the ADH and ALDH activities in the MCP-M group increased by 52.30% and 143.92%, respectively, while the same dose of OP boosted ADH and ALDH activities by 39.14% and 78.96%, respectively. These results indicate that MCPs can promote the metabolism of alcohol and acetaldehyde in the liver and that the effect is more significant than that of OP.

### 3.5. MCPs Improve Oxidative Stress in ALD Mice

**Figure 3:**
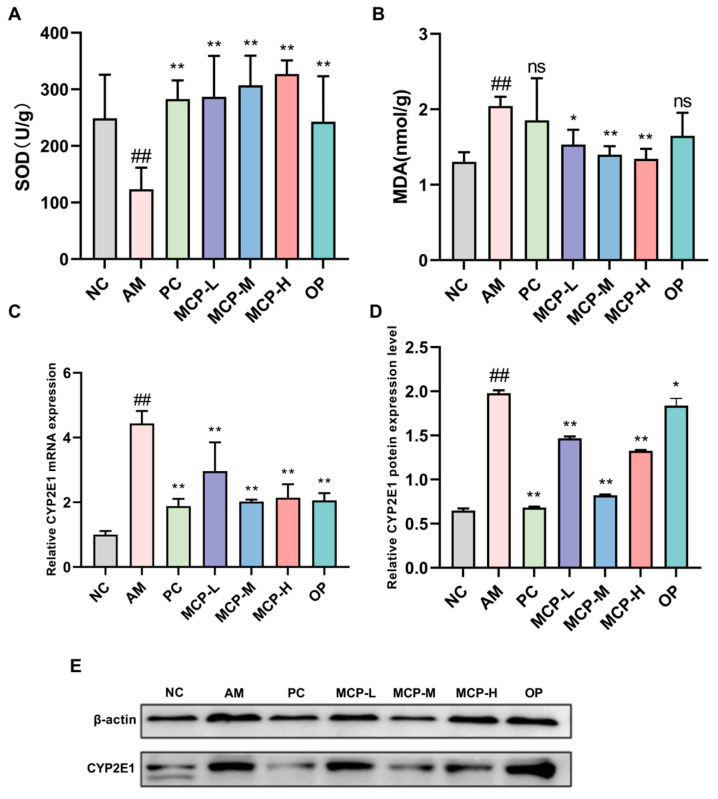
Effects of MCPs on SOD (**A**) and MDA (**B**) levels as well as CYP2E1 mRNA (**C**) and protein (**D**,**E**) expression in the liver. Data are expressed as the means ± SEM (n = 10). Compared with the NC group: ## *p* < 0.01; compared with the AM group: * *p* < 0.05, ** *p* < 0.01 and ns *p* > 0.05.

Oxidative stress resulting from the metabolism of alcohol is considered a significant factor in the development of alcohol-induced liver damage [25]. To investigate the impact of MCPs on alcohol-induced liver oxidative stress injury in mice, the levels of SOD and MDA in the liver, along with the expression of CYP2E1, were measured. As shown in Figure 3A,B, SOD activity was significantly lower and MDA content was significantly higher (*p* < 0.01) in the AM group compared with the NC group, indicating heightened oxidative stress due to alcohol consumption. However, pretreatment with MCPs led to notable improvements. Specifically, the MCP-H group showed a 165.01% increase in SOD activity and a 52.21% drop in MDA content compared to the AM group. These findings revealed that MCPs could mitigate alcohol-induced oxidative stress injury in the liver by enhancing SOD activity and reducing MDA production. The hepatic expression level of CYP2E1 was detected to explore the effect of MCP pretreatment on the expression of genes and proteins associated with alcohol-induced oxidative stress. Compared to the NC group, the expression levels of CYP2E1 mRNA and protein were notably elevated in the AM group. MCPs exhibited hepatoprotective properties against alcohol-induced oxidative stress by suppressing CYP2E1 expression in the liver (Figure 3C–E). The results suggested that the protective effect of MCPs against acute alcohol-induced liver injury may reduce oxidative stress by down-regulating the expression of CYP2E1.

### 3.6. MCPs Improve Abnormal Lipid Metabolism in ALD Mice

**Figure 4:**
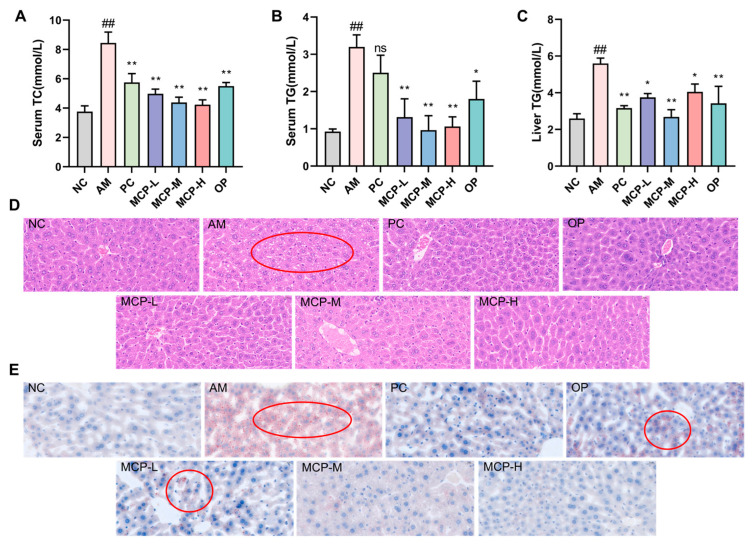
MCP-attenuated lipid accumulation in sera and livers of ALD mice. (**A**) Serum TC; (**B**) serum TG; (**C**) liver TG; (**D**) H&E staining (100× magnification); (**E**) Oil Red O staining (100× magnification). Data are expressed as the means ± SEM (n = 10). Compared with the NC group: ## *p* < 0.01; compared with the AM group: * *p* < 0.05 ** *p* < 0.01 and ns *p* > 0.05.

TG and TC are crucial indicators of lipid metabolism in the body, and hepatic steatosis or disorders of hepatic lipid metabolism can lead to elevated TG and TC levels. As illustrated in Figure 4A,B, serum TC and TG levels were considerably greater in the AM group than in the NC group (*p* < 0.01). The MCPs considerably prevented the increase in serum TC and TG levels (*p* < 0.01), an effect that was superior to that of the PC and OP. Similarly, liver TG levels were notably higher in the AM group than in the NC group (Figure 4C). MCP pretreatment resulted in a significant decrease in hepatic TG levels compared with the AM group. The MCP-M group showed the most significant improvement, and the levels were even brought back to normal.

The results of the HE staining (Figure 4D) revealed that the hepatocytes of mice in the AM group displayed severe pathological alterations, including disorganized hepatocyte arrangement and enlarged cells with irregular fat vacuoles, indicating acute alcohol-induced severe liver injury. Following intervention with the MCP, OP, and PC, the liver cells of mice exhibited clear boundaries, although a few fat vacuoles remained in the PC group. In comparison to the AM group, the MCP group demonstrated a dose-dependent reduction in hepatocyte injury, with the MCP-H group showing a restored hepatocyte morphology similar to that of the NC group. The Oil Red O staining results (Figure 4E) were consistent with the HE staining results. The hepatocytes of the AM group contained numerous red fat droplets, while a dose-dependent reduction in red fat droplets was observed in all MCP dose groups. The hepatocyte morphology in the MCP-H group closely resembled that of the NC group.

**Figure 5:**
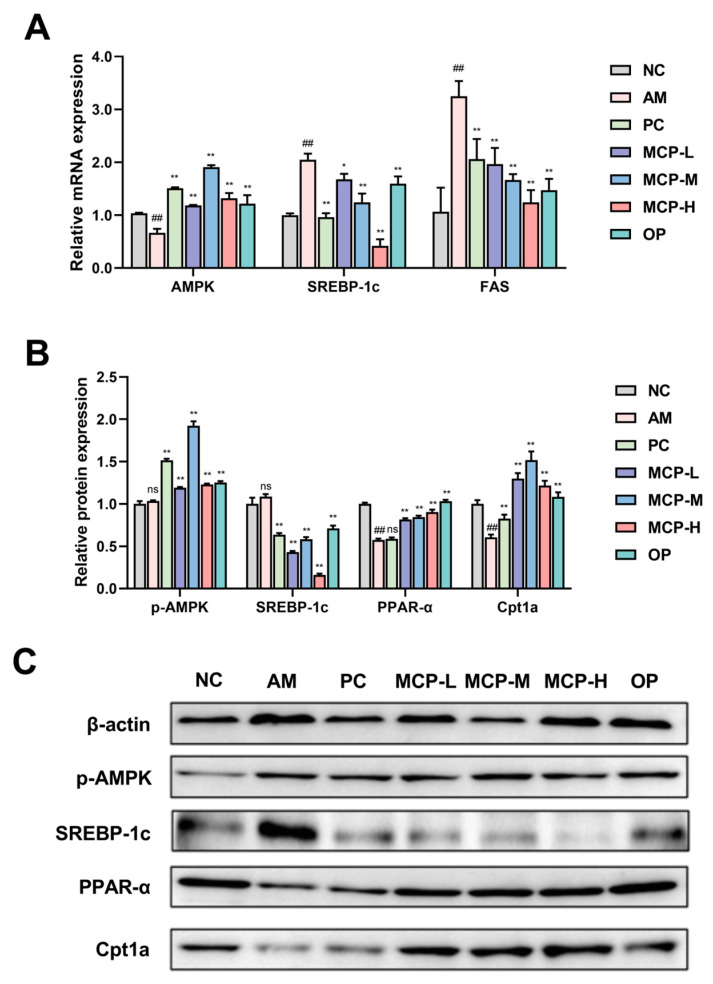
Effects of MCP treatment on the expression of mRNA (**A**) and protein (**B**,**C**). Data are expressed as the means ± SEM (n = 10). Compared with the NC group: ## *p* < 0.01; compared with the AM group: * *p* < 0.05, ** *p* < 0.01 and ns *p* > 0.05.

In order to explore the potential molecular mechanism of MCPs in regulating lipid metabolism, the expression of genes and proteins involved in lipid metabolism in the liver was examined by the qRT-PCR and Western blot methods (Figure 5). Given the pivotal role of the AMPK signaling pathway in lipid metabolism, we investigated whether the mitigating effect of MCPs on acute alcohol-induced liver injury was associated with the expression of AMPK and its downstream signaling pathway targets, including SREBP-1c, FAS, PPAR-α, and Cpt1a. In comparison to the NC group, the expression levels of AMPK mRNA and p-AMPK protein in the liver of mice were decreased in the AM group, resulting in a notable increase in the expression levels of SREBP-1c and FAS mRNA, and a significant decrease in the expression levels of PPARα and Cpt1a proteins. MCP pretreatment increased the expression levels of AMPK mRNA and p-AMPK protein and inhibited SREBP-1c and FAS mRNA expression significantly. At the same time, it facilitated a significant elevation of PPARα and Cpt1a protein expression levels.

### 3.7. MCPs Improve Inflammatory Response in ALD Mice

**Figure 6:**
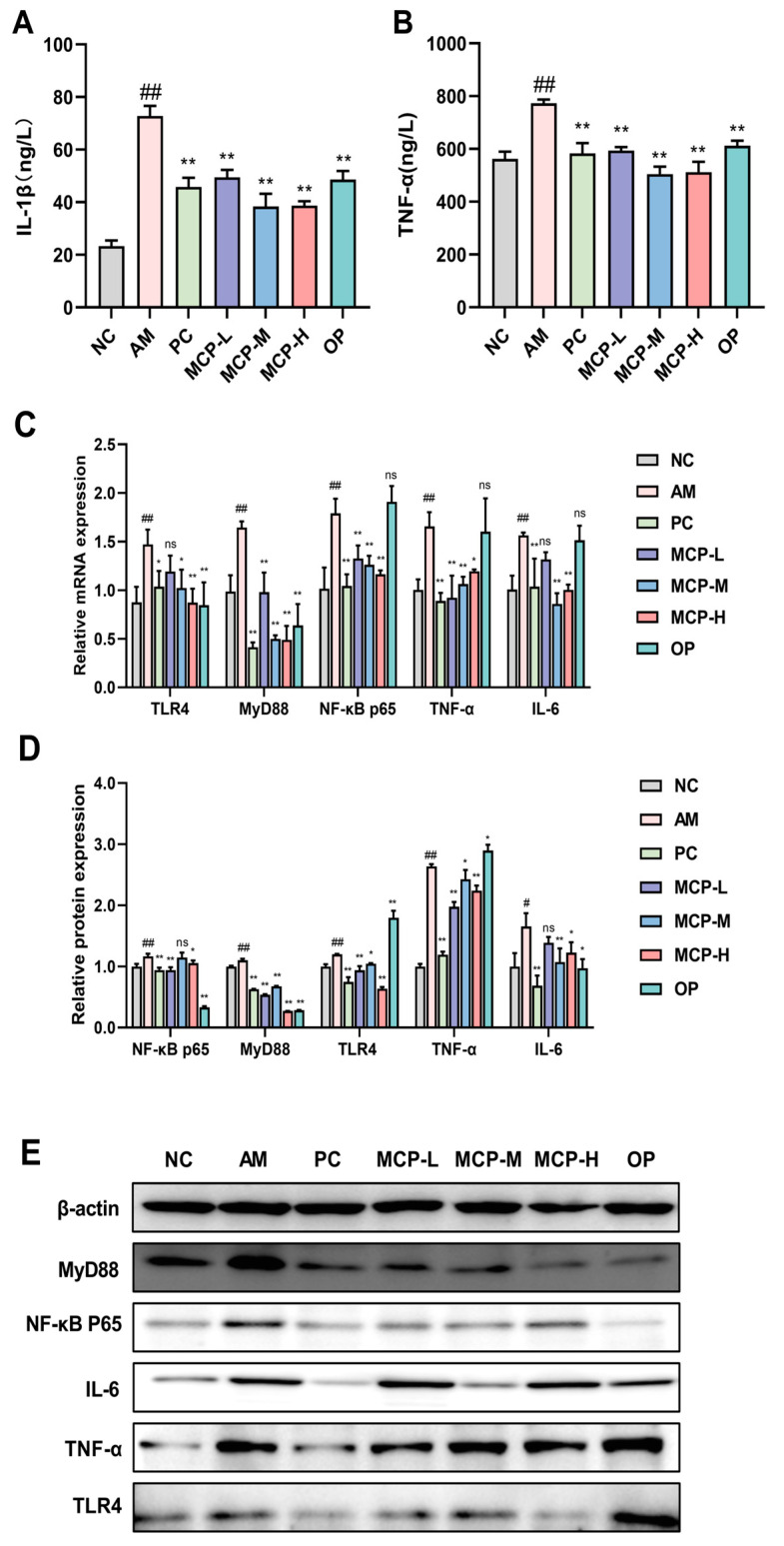
Effect of MCPs on inflammatory response in ALD mice. (**A**) Serum IL-1β level; (**B**) serum TNF-α level; (**C**) the expression of mRNA; (**D**,**E**) the expression of protein. Data are expressed as the means ± SEM (n = 10). Compared with the NC group: # *p* < 0.05 and ## *p* < 0.01; compared with the AM group: * *p* < 0.05, ** *p* < 0.01 and ns *p* > 0.05.

The potential protective effect of MCPs against acute alcohol-induced liver injury was further investigated by detecting the levels of inflammatory factors. The effect of MCPs on serum cytokine levels in mice is depicted in Figure 6A,B. The levels of IL-1β and TNF-α in the AM group were significantly higher than those in the NC group (*p* < 0.01), indicating an imbalance in the body’s inflammatory system due to acute alcohol attack. However, MCP pretreatment inhibited this increase (*p* < 0.01), and, in particular, the levels of TNF-α returned to normal. Moreover, the MCPs were more effective in improving the secretion levels of pro-inflammatory cytokines than was observed in the PC and OP groups.

The impact of MCP pretreatment on the expression of several hepatic inflammatory genes and proteins was assessed. Results from qRT-PCR (Figure 6C) and Western blotting (Figure 6D,E) indicated a notable rise in the mRNA and protein expression levels of TLR4, MyD88, NF-κB P65, TNF-α, and IL-6 in mice with acute alcoholic liver injury (*p* < 0.01 or *p* < 0.05). However, the expression levels of TLR4, MyD88, NF-κB p65, TNF-α, and IL-6 were found to be reversed after MCP pretreatment.

### 3.8. MCPs Improve Gut Microbiota Composition in ALD Mice

**Figure 7:**
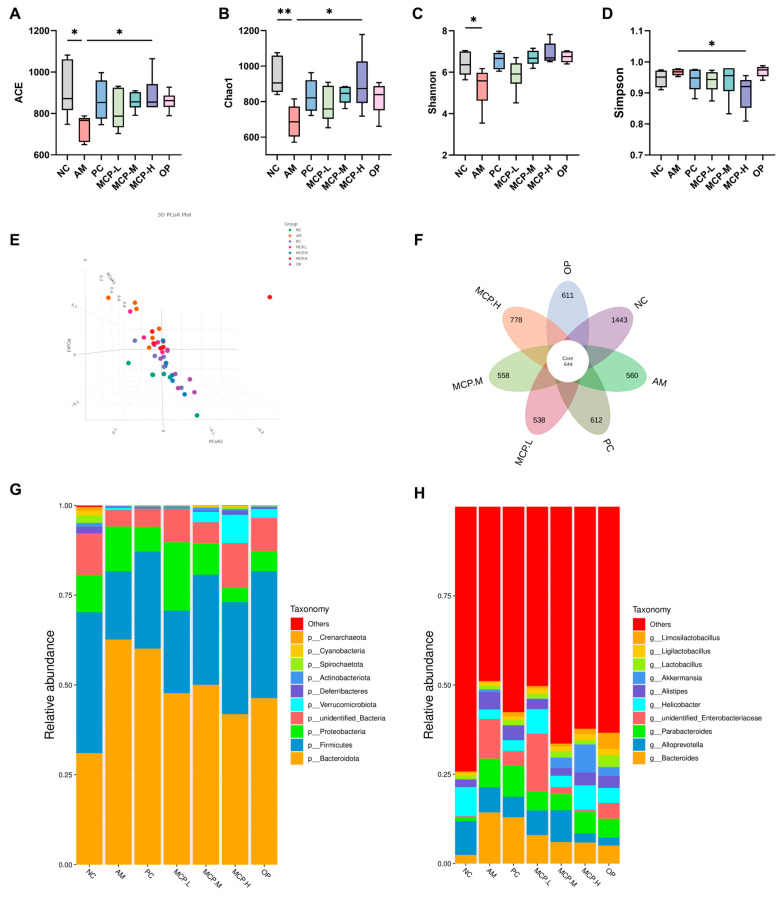
Regulatory effect of MCPs on intestinal microecology in ALD mice. (**A**) ACE index; (**B**) Chao1 index; (**C**) Shannon index; (**D**) Simpson index; (**E**) PCoA analysis; (**F**) the number of ASVs; relative abundance of species in each group at the phylum (**G**) and genus (**H**) levels. Data are expressed as the means ± SEM (n = 10). Compared with the AM group: * *p* < 0.05 and ** *p* < 0.01.

Currently, numerous scholars both domestically and internationally assert that the development of ALD is intricately linked to the dysbiosis of gut microbiota. Under normal physiological conditions, the composition of gut microbiota maintains a symbiotic equilibrium within the human body, and prolonged excessive alcohol consumption may disrupt this balance. Therefore, we investigated the impact of MCPs on microbial diversity and composition. The α-diversity of the gut microbiota in ALD mice was determined. The results showed that compared to the NC group, the ACE (Figure 7A), Chao1 (Figure 7B), Shannon (Figure 7C), and Simpson (Figure 7D) indices significantly decreased in the AM group, indicating that alcohol can lead to bacterial overgrowth and reduce the diversity of the gut microbiota, but these changes can be reversed with MCP intervention. These results suggest that MCP pretreatment significantly restores the richness and diversity of the gut microbiota in ALD mice. In addition, β-diversity was used to analyze overall structural changes in the gut microbiota. The results showed significant changes in the overall structure of the gut microbiota in ALD mice, with the MCP group moving closer to the NC group and showing some overlap (Figure 7E). A denoising analysis based on effective data generated Amplicon Sequence Variants (ASVs), and the high-dose MCP group significantly restored the microbial abundance in the gut (Figure 7F). These results indicate that MCP pretreatment can restore the stability of the gut microbiota in ALD mice and has the ability to regulate microbial community structure.

Further analysis revealed detailed differences in the relative abundance of bacterial communities at the phylum and genus levels. Figure 7G primarily displays the nine most abundant phyla in the gut microbiota of ALD mice, with *Bacteroidota*, *Firmicutes*, and *Proteobacteria* being the most dominant. Compared to the AM group, all doses of MCPs reduced the abundance of *Bacteroidota* and *Proteobacteria*, while increasing the abundance of *Firmicutes*. Subsequently, differences between groups were determined at the genus level (Figure 7H). Compared to the AM group, the abundances of the *Bacteroides*, *Parabacteroides*, and *Alistipes* genera decreased in all the MCP groups, while the relative abundance of *Alloprevotella*, *Helicobacter*, and *Akkermansia* genera increased. These results indicate that MCP pretreatment can improve gut dysbiosis, promote the proliferation of beneficial gut bacteria, and improve alcohol-induced intestinal barrier dysfunction.

## 4. Discussion

Alcohol consumption is a common part of social gatherings and celebrations. However, excessive and prolonged alcohol intake can lead to serious health issues, with alcoholic liver damage being one of the most significant [26]. In recent years, there has been growing interest in the role of food in preventing and mitigating the effects of alcohol on the liver. Bioactive peptides extracted from food not only reduce oxidative stress but also possess anti-inflammatory and anti-liver fibrosis properties. Moreover, compared to medications that may cause adverse reactions or other toxicities, these peptides have higher nutritional and safety characteristics [27]. Hence, our study aimed to explore the hepatoprotective effect of MCPs on mice with acute alcohol-induced liver injury, as well as its underlying mechanism.

The biological activity of bioactive peptides is intimately correlated with their molecular weight and amino acid composition. Low molecular weight bioactive peptides are more hepatoprotective than large molecular weight peptides because they may cross the intestinal barrier more readily and show improved bioactivity [28]. The content of small and medium molecular peptides (MW < 1 kDa) in the MCPs was 94.25%. HPLC analysis showed that the content of essential amino acids in the MCPs reached 42.98%, and the proportion of branched-chain amino acids in the MCPs was 19.90%. Consuming branched-chain amino acids is considered a useful strategy for alleviating liver injury [29]. In addition, MCPs rich in hydrophobic amino acids (such as Gly, Ala, Val, Met, Ile, Leu, Phe, and Pro) helped to form more hydrophobic peptides to enhance their free radical scavenging ability. Amino acid composition has been reported to influence the hepatoprotective properties of bioactive peptides [30]. For example, specific amino acids, including Ala, Leu, Pro, His, and Lys, have been proven to produce stable NAD+ in the tricarboxylic acid cycle in vivo [31,32]. Therefore, the results suggested that MCPs containing the aforementioned amino acids may contribute to NAD+ production and subsequently contribute to enhanced alcohol metabolism. Similar results were found in glycosylated zein peptides [33].

Excessive ethanol intake in mice leads to a gradual onset of symptoms, such as with their hind legs, sluggish action, lethargy, and LORR [34]. The disappearance of the LORR serves as a criterion for whether the mice are intoxicated or not. In the present study, an acute single administration of 600 mg/kg·bw of ethanol induced hypnotic effects in mice. However, pretreatment with MCPs significantly ameliorated the condition, as evidenced by the reduced LORR rate, extended LORR latency, and decreased LORR duration. In normal hepatocytes, ALT and AST are mainly distributed in mitochondria and the cytoplasm. When hepatocytes are damaged, intracellular ALT and AST will flow into the blood through the cell membrane. Therefore, the activity level of ALT and AST in serum can reflect the extent of liver cell damage [35]. Acute alcoholism can cause liver injury, and the activity levels of ALT and AST in serum are negatively correlated with liver injury [36]. The increase in serum AST and ALT levels in the current research was considerably suppressed by MCP pretreatment, indicating that MCPs may have a protective role against alcoholic liver impairment. After alcohol is absorbed into the circulation, it is oxidized to acetaldehyde primarily in the liver by ADH. Acetaldehyde is subsequently further metabolized to harmless acetic acid by ALDH, with water and carbon dioxide excreted from the body [37,38]. Nevertheless, acute alcohol consumption could strongly inhibit liver ADH activity and thus lower the rate of alcohol metabolism [39]. The findings of the present study showed that MCP pretreatment markedly raised the activities of hepatic ADH and ALDH.

When the concentration of ethanol in the bloodstream exceeds a certain level, it leads to the production of cytochrome P450 oxidase (CYP450) in liver cells, which in turn activates CYP2E1 to generate a significant amount of reactive oxygen species (ROS) [40]. The accumulation of ROS intensifies oxidative stress in liver cells. Excessive oxidative damage can cause lipid peroxidation, leading to an increase in the content of MDA, the end product of the peroxidation reaction, and accelerating the depletion of SOD [41]. SOD is a highly efficient antioxidant enzyme found in animals. Its main function is to eliminate free radicals within the body and prevent them from causing harm [42]. MDA is a primary reactive aldehyde produced in biological membranes as a result of polyunsaturated fatty acid peroxidation caused by ROS. Therefore, MDA content is a crucial marker for measuring the level of lipid peroxidation in the body, thereby reflecting the severity of liver damage resulting from an attack by free radicals [43]. In the present study, SOD activity was significantly lower while MDA content was considerably higher in the livers of mice in the AM group than in the NC group. This indicated that acute alcohol intake resulted in the depletion of antioxidant enzyme activity and the deepening of lipid peroxidation in the liver tissues of mice. However, pretreatment with MCPs significantly increased SOD activity and decreased MDA elevation even at low dose levels. Previous research reported that activation of the CYP2E1 protein is highly associated with oxidative stress [44]. Notably, MCP intervention effectively suppressed CYP2E1 mRNA and protein expression in mice, suggesting that MCPs may alleviate acute alcohol-induced liver injury by ameliorating oxidative stress. Hydrophobic amino acid residues like Leu, Phe, Pro, and Val have been reported to have antioxidant activity. This means that they may increase the solubility of peptides in lipids and accelerate the scavenging of fat-soluble free radicals [45]. From this standpoint, the decrease in oxidative stress in the MCP group could be due to the presence of hydrophobic amino acids and peptides containing these amino acids.

Alcohol-induced liver injury often presents with alcoholic fatty liver as its initial and prevalent manifestation, marked by the build-up of triglycerides within liver cells. The metabolism of ethanol and acetaldehyde results in the generation of elevated levels of NADH, causing a disruption in the cellular redox balance and subsequently promoting excessive synthesis of fatty acids and triglycerides [46]. SREBP-1c controls the lipogenic pathway and regulates the expression of the related target FAS. The overexpression of SREBP-1c enhances fatty acid and TG synthesis and the abnormal accumulation of hepatic TG [47,48]. AMPK serves as a regulator for both anabolic and catabolic pathways. Activated AMPK decreases lipogenesis in the liver by inhibiting SREBP-1c expression [49,50]. PPAR-α is a nuclear hormone receptor involved in the regulation of lipid metabolism and inflammation. PPAR-α induces the expression of Cpt1a, the rate-limiting enzyme for mitochondrial fatty acid oxidation in the liver. It has been documented that the suppression of PPAR-α and Cpt1a protein function can diminish the capacity of fatty acid β-oxidation to impede TG synthesis and lipid accumulation [51,52]. In this investigation, the consumption of alcohol in mice resulted in an increase in serum and liver levels of TG and TC. However, MCP pretreatment significantly prevented this elevation. Furthermore, MCPs inhibited the activation of SREBP-1c and FAS, leading to a reduction in lipid synthesis and hepatic lipogenesis in mice. Additionally, the MCPs increased the expression of p-AMPK, PPAR-α, and Cpt1a proteins in the livers, which enhanced enzyme activities and promoted the oxidative decomposition of fatty acids. These findings suggest that MCPs have the potential to improve metabolic disorders associated with acute alcohol-induced liver injury by activating the AMPK/SREBP-1c/FAS and PPAR-α/Cpt1a signaling pathways.

Long-term accumulation of fat exacerbates liver cell inflammation, which is another key pathogenic mechanism of alcohol-induced liver injury. Acute alcohol consumption significantly increases the levels of the pro-inflammatory cytokines IL-1β and TNF-α, consistent with previous reports [53]. Interestingly, all doses of the MCPs significantly reduced the levels of pro-inflammatory cytokines. Abnormal signaling pathways related to alcohol-induced liver injury inflammation may be the direct cause of the inflammatory response [54,55]. Current research indicates that alcohol misuse impairs the function of the intestinal epithelial barrier, increasing permeability and facilitating the transfer of endotoxins into the portal vein and circulation into the liver. Kupffer cells are activated by endotoxins that enter the liver through the TLR4/NFκB-dependent mechanism. MyD88 is a crucial adaptor protein in the TLR4 signaling pathway that activates NF-κB and the expression of pro-inflammatory cytokine target genes. Previous studies have demonstrated the important role of the TLR4/MyD88/NF-κB pathway in liver inflammation. TLR4 can activate NF-κB through MyD88-dependent and independent signaling pathways, promoting the release of various inflammatory factors [56,57]. The results of this study showed that MCP pre-treatment significantly lowered the expression levels of TLR4, MyD88, NF-κB P65, TNF-α, and IL-6. This indicates that the MCPs reduced the levels of the downstream inflammatory factors TNF-α and IL-1β by suppressing the TLR4/MyD88/NF-κB signaling pathway, thereby alleviating inflammation-induced liver injury.

Numerous studies have shown that long-term exposure to alcohol can lead to changes in the composition of the gut microbiota, promoting the overgrowth of pathogenic bacteria, inhibiting beneficial bacteria in the gut, and significantly increasing the harmful metabolites entering the liver [58,59]. In this study, compared to the AM group, the gut microbiota of mice pre-treated with MCPs showed a significant increase in species diversity and quantity, indicating that MCPs can effectively restore gut microbiota diversity. Based on the distribution at the phylum and genus levels and the significant differences in species, it can be concluded that MCP pretreatment can improve gut dysbiosis to varying degrees, promoting an increase in the relative abundance of beneficial bacteria such as *Alloprevotella* and *Akkermansia* in the gut, and improving alcohol-induced intestinal barrier dysfunction.

## 5. Conclusions

In this study, we extracted MCPs from *Mactra chinenesis* and investigated their preventive effects on alcohol-induced liver injury and the underlying mechanisms in an ALD mouse model. The results indicated that MCPs could accelerate alcohol metabolism, inhibit lipid peroxidation, alleviate liver tissue inflammation, and improve gut microbiota dysbiosis, thereby protecting the liver from acute alcohol-induced damage. The findings of this study support the potential of MCPs as protective agents against hangover and liver injury, as well as a natural source of functional food. However, further detailed investigations are still needed, including studying their cytotoxicity effects by clinical trials and interactions with the gastrointestinal tract, before practical utilization can begin.

## Figures and Tables

**Table 1:** The molecular weight distribution of extracted peptides.

Molecular Weight (Da)	MCPs (%)	OP (%)
>10,000	0.54	0.04
10,000~5000	0.13	0.05
5000~3000	0.30	0.27
3000~2000	0.61	0.76
2000~1000	4.15	6.63
1000~500	20.04	21.98
500~180	62.01	53.66
<180	12.20	16.60

**Table 2:** Amino acid compositions of extracted peptides (n = 3).

Amino Acids	Content (g/100 g)	
	MCP	OP
Asp	2.68 ± 0.12	2.75 ± 0.08
Glu	16.72 ± 0.51	14.01 ± 0.48
Ser	2.51 ± 0.08	1.96 ± 0.11
His ^4^	1.51 ± 0.13	1.70 ± 0.09
Gly ^3^	8.86 ± 0.55	9.69 ± 0.81
Thr ^1^	4.35 ± 0.02	3.01 ± 0.17
Arg	10.03 ± 0.71	13.09 ± 1.03
Ala ^3^	9.36 ± 0.69	8.25 ± 0.16
Tyr ^4^	4.18 ± 0.10	4.84 ± 0.13
Cys	0.50 ± 0.12	0.26 ± 0.08
Val ^1,2,3^	3.18 ± 0.12	3.40 ± 0.11
Met ^1,3,4^	6.19 ± 0.49	3.53 ± 0.24
Phe ^1,3^	5.35 ± 0.38	5.76 ± 0.16
Ile ^1,2,3^	3.01 ± 0.12	3.01 ± 0.20
Leu ^1,2,3^	13.71 ± 0.83	12.17 ± 0.96
Lys ^1,4^	7.19 ± 0.64	11.13 ± 0.77
Pro ^3^	0.84 ± 0.13	1.31 ± 0.20
Essential amino acid	42.98	42.01
Branched chain amino acid	19.90	18.58
Hydrophobic amino acids	50.50	47.12

^1^ Essential amino acid; ^2^ Branch-chain amino acids; ^3^ Hydrophobic amino acids; ^4^ Antioxidant amino acids.

**Table 3:** Effects of MCPs on the LORR, body weight, liver weight, and liver index in mice.

Treatment Groups	LORR Rate (%)	Latency of LORR (min)	Duration of LORR (min)	Body Weight (g)	Liver Weight (g)	Liver Index (%)
NC	-	-	-	44.75 ± 3.32 ^a^	1.685 ± 0.228 ^b^	3.735 ± 0.451 ^g^
AM	80	29.39 ± 21.71 ^f^	434.83 ± 99.42 ^a^	37.99 ± 6.09 ^c^	1.691 ± 0.181 ^a^	4.175 ± 0.193 ^a^
PC	70	30.70 ± 30.30 ^e^	316.14 ± 100.06 ^e^	38.61 ± 2.86 ^bc^	1.511 ± 0.196 ^g^	3.881 ± 0.374 ^e^
OP	70	35.21 ± 11.63 ^c^	318.85 ± 82.60 ^d^	39.04 ± 4.97 ^bc^	1.574 ± 0.283 ^e^	3.939 ± 0.583 ^c^
MCP-L	60	30.83 ± 10.45 ^d^	334.24 ± 45.67 ^b^	38.66 ± 3.07 ^bc^	1.603 ± 0.175 ^d^	4.054 ± 0.246 ^b^
MCP-M	60	37.54 ± 12.57 ^b^	321.33 ± 85.57 ^c^	37.62 ± 4.97 ^c^	1.641 ± 0.201 ^c^	3.936 ± 0.229 ^d^
MCP-H	60	42.21 ± 15.61 ^a^	312.40 ± 26.15 ^f^	40.52 ± 3.11 ^b^	1.549 ± 0.159 ^f^	3.814 ± 0.271 ^f^

Data are expressed as the means ± SEM (n = 10). Values with different letters are significantly different (*p* < 0.05).

## Data Availability

The original contributions presented in the study are included in the article/Supplementary Materials, further inquiries can be directed to the corresponding authors.

## Supplementary Materials

The following supporting information can be downloaded at: https://www.mdpi.com/article/10.3390/foods13101431/s1, Figure S1: HPLC of molecular weight distribution; Figure S2: Amino acid chromatogram; Table S1: List of abbreviations; Table S2: Primers information for PCR.
