# Supplementary material for: Effects of Mactra chinenesis Peptides on Alcohol-Induced Acute Liver Injury and Intestinal Flora in Mice
Source: Foods. 2024 May 7;13(10):1431. doi: 10.3390/foods13101431 (PMC11119424; doi:10.3390/foods13101431)
Supplement: Supplementary file 1 [file foods-13-01431-s001.zip › foods-2959223-supplementary.pdf]

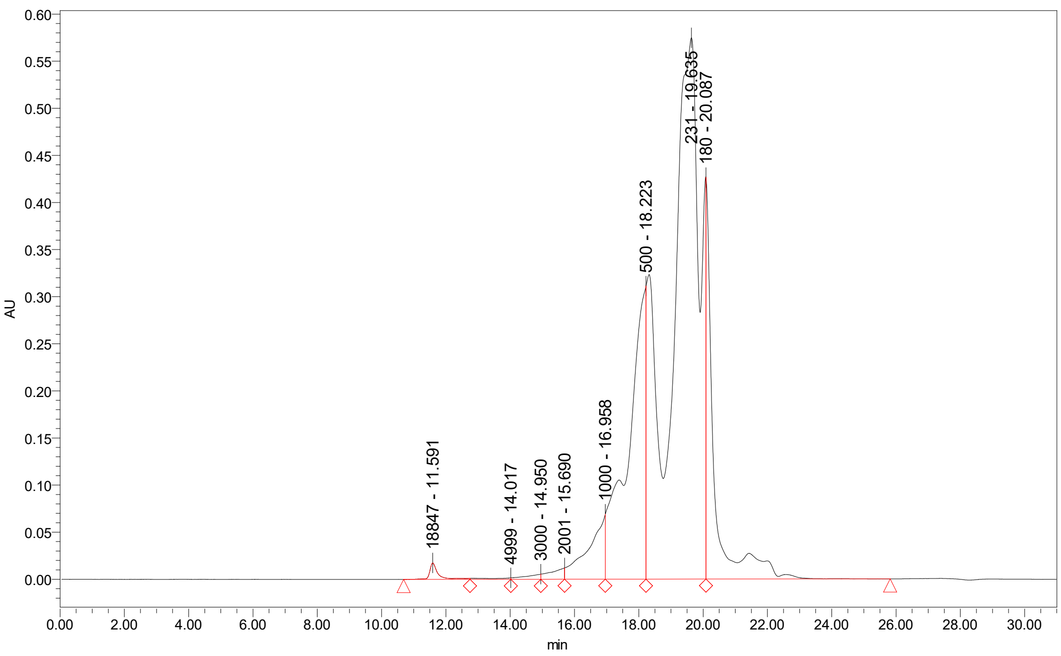

Figure. S1 HPLC of molecular weight distribution

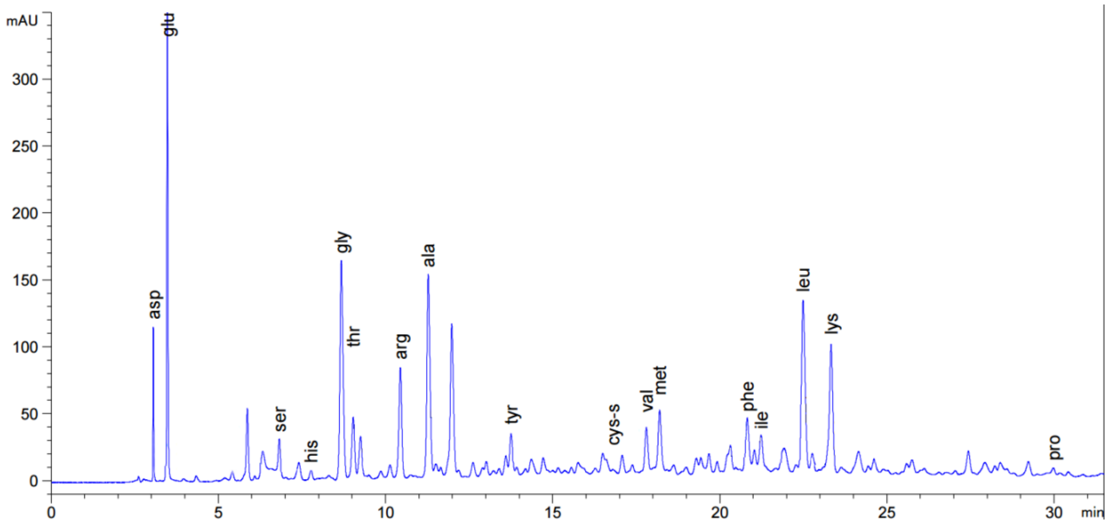

Figure S2. Amino acid chromatogram

Table S1. List of abbreviations.

NO.	Name	Abbreviation
1	Mactra chinenesis	MC
2	Mactra chinenesis peptides	MCP
3	High performance liquid chromatography	HPLC
4	Real-time fluorescence quantitative polymerase chain reaction	qRT-PCR
5	Alcohol dehydrogenase	ADH
6	Aldehyde dehydrogenase	ALDH
7	Total cholesterol	TC
8	Triglyceride	TG
9	Alanine aminotransferase	ALT
10	Aspartate aminotransferase	AST
11	Superoxide dismutase	SOD
12	Malondialdehyde	MDA
13	Interleukin-1β	IL-1β
14	Tumor necrosis factor-α	TNF-α
15	(AMP)-activated protein kinase	p-AMPK
16	Toll-like receptor 4	TLR4
17	Cytochrome P450 2E1	CYP2E1
18	Myeloid differential protein-88	MyD88
19	Peroxisome proliferator-activated receptor-α	PPAR-α
20	Carnitine palmitoyltransferase 1a	Cpt1a
21	Sterol regulatory elementbinding protein 1c	SREBP-1c
22	Interleukin-6	IL-6
23	Specific pathogen-free	SPF
24	Normal control	NC
25	Alcohol-induced model	AM
26	Positive control	PC
27	Oyster peptide	OP
28	Low dose of MCP	MCP-L
29	Median dose of MCP	MCP-M
30	High dose of MCP	MCP-H
31	Loss of righting reflex	LORR
32	Hematoxylin and eosin	H&E
33	Amplicon Sequence Variants	ASVs
34	Reactive oxygen species	ROS

Table S2. Primers information for PCR.

Gene	Forward(5′-3′)	Reverse (5′-3′)
CYP2E1	CGGGATGTGACTGACTGTCTCC	AGTTGTGCTGGTGGTCTCTGTTC
AMPK	ATGATGAGGTGGTGGAGCAGAGG	GGTTCTCGGCTGTGCTGGAATC
SREBP-1c	TCCACCATCGGCACCCACTG	GGCACTGGCTCCTCTTTGATTCC
FAS	TGCTTGCTGGCTCACAGTTAAGAG	TTCACGAACCCGCCTCCTCAG
TLR4	CCGCTTTCACCTCTGCCTTCAC	ACCACAATAACCTTCCGGCTCTTG
MyD88	GCAGAACCAGGAGTCCGAGAAG	GATGCCTCCCAGTTCCTTTGTTTG
NF-κB P65	TCGAGTCTCCATGCAGCTACGG	CGGTGGCGATCATCTGTGTCTG
TNF-α	CCACCACGCTCTTCTGTCTACTG	TGGTTTGTGAGTGTGAGGGTCTG
IL-6	TTCTTGGGACTGATGCTGGTGAC	CTGTTGGGAGTGGTATCCTCTGTG
